# Dual Targeting of FAP-Directed Nanoparticles and FRα-Specific CAR-T Cells Induces Additive Anti-Tumor Effects in Triple-Negative Breast Cancer

**DOI:** 10.7150/ijbs.122417

**Published:** 2026-02-18

**Authors:** Tanva Thongkleang, Suyanee Thongchot, Kamonlatth Rodponthukwaji, Piriya Luangwattananun, Kwanruthai Tadpetch, Pa-thai Yenchitsomanus, Peti Thuwajit, Primana Punnakitikashem, Chanitra Thuwajit

**Affiliations:** 1 Department of Immunology, Faculty of Medicine Siriraj Hospital, Mahidol University, Bangkok, Thailand.; 2 Siriraj Center of Research Excellence for Cancer Immunotherapy, Research Department, Faculty of Medicine Siriraj Hospital, Mahidol University, Bangkok, Thailand.; 3 Department of Biochemistry, Faculty of Medicine Siriraj Hospital, Mahidol University, Bangkok, Thailand.; 4 Siriraj Center of Research Excellence in Theranostic Nanomedicine, Faculty of Medicine Siriraj Hospital, Mahidol University, Bangkok, Thailand.; 5 Division of Molecular Medicine, Research Department, Faculty of Medicine Siriraj Hospital, Mahidol University, Bangkok, Thailand.; 6 Division of Physical Science and Center of Excellence for Innovation in Chemistry, Faculty of Science, Prince of Songkla University, Hat Yai, Songkhla, Thailand.

**Keywords:** Cancer-associated fibroblast, Chimeric antigen receptor-T cell, Fibroblast activation protein alpha, Folate receptor alpha, Nanoparticle, Triple-negative breast cancer

## Abstract

Triple-negative breast cancer (TNBC) is an aggressive malignancy with limited treatment options. It lacks hormone receptors and human epidermal growth factor receptor 2. The immunosuppressive tumor microenvironment (TME), particularly cancer-associated fibroblasts (CAFs), significantly hinders chimeric antigen receptor (CAR)-T cell therapy success. Novel strategies to overcome TME-mediated immunosuppression are urgently needed. We evaluated whether targeting CAFs with fibroblast activation protein alpha (FAP)-coated, 8-*O*-methylfusarubin-loaded nanoparticles called anti-FAP@OMF-NPs could enhance the anti-tumor efficacy of folate receptor alpha (FRα)-specific CAR-T cells against TNBC in a 3D cancer cells-CAFs co-culture heterospheriod (HS) model. FRα and FAP expression in TNBC cells and primary breast CAFs were assessed using immunofluorescence and flow cytometry. Anti-FRα-CAR-T cells were generated via lentiviral transduction and characterised for activation markers. Cytotoxic activity of CAR-T cells, anti-FAP@OMF-NPs, and their combination was evaluated in 3D-HS comprising FRα-high TNBC cells and FAP-high CAFs. A fluorescent transfection assay measured cell viability. Cytokine bead arrays quantified IFN-γ, granzyme A, and granzyme B levels to assess anti-tumor immune activation. PC-B-130CAFs and PC-B-132CAFs demonstrated high FAP expression compared with PC-B-004CAFs and normal human dermal fibroblast cells (HDFa). Anti-FRα-CAR-T cells selectively targeted FRα-positive TNBC cells whilst showing minimal cytotoxicity towards normal MCF-10A cells. Anti-FAP@OMF-NPs induced potent cytotoxic effects specifically in FAP-expressing CAFs. Combined treatment significantly enhanced the destruction of MDA-MB-231/130CAF and MDA-MB-231/132CAF HSs compared with monotherapies. This combination increased secretion of IFN-γ, granzyme A, and granzyme B from anti-FRα-CAR-T cells. Targeting CAFs using anti-FAP@OMF-NPs enhances the cytotoxic efficacy of FRα-specific CAR-T cells in TNBC. This combinatorial approach offers a promising strategy to overcome TME-mediated immunosuppression. These findings support further development of dual-targeting approaches to improve therapeutic outcomes in TNBC.

## Introduction

Breast cancer is the most common cause of cancer-related death worldwide 1. Based on expression of estrogen receptor (ER), progesterone receptor (PR), and human epidermal growth factor receptor 2 (HER2), together with the proliferative marker Ki-67, breast cancer can be classified into 4 subtypes 2, 3. These subtypes are luminal A, luminal B, HER2-overexpressed, and triple-negative breast cancer (TNBC). TNBC exhibits the worst prognosis and most limited treatment options due to the absence of ER, PR, and HER2 4, 5. Consequently, hormone therapy and HER2-targeted therapy provide no benefit. Patients diagnosed with TNBC have a higher risk of metastasis within the first 3 years of primary diagnosis 6. Fewer than 30% of patients diagnosed with metastatic TNBC survive beyond 5 years 7. Although chemotherapy remains the standard of care for TNBC, it causes systemic toxicity owing to non-specific drug reactions. Therefore, novel therapeutic approaches that specifically target cancer cells whilst minimising cytotoxicity to normal cells must be developed.

Adoptive cell transfer (ACT) is a personalised cancer immunotherapy that utilises the administration of tumor-reactive T lymphocytes derived from the patient's blood to eradicate tumor cells 8. Chimeric antigen receptor-T cell (CAR-T cell) therapy is one form of ACT. T cells are engineered to express chimeric antigen receptors (CARs) to recognise and eliminate tumor-associated antigen (TAA). Several studies have reported successful clinical trials of CAR-T cell therapy in haematological cancers. This success led to US Food and Drug Administration (FDA) approval of CAR-T cell products for treating haematological malignancies 9. However, CAR-T cells face significant challenges in treating solid tumors, although numerous antigenic targets have been validated 10. Folate receptor alpha (FRα) is a membrane-bound protein with limited expression in normal cells but high expression in several tumors, including TNBC 11. FRα expression occurs in approximately 60% of TNBC samples and correlates with cancer cell proliferation and tumor progression 12. This expression pattern highlights its value in cellular immunotherapy for TNBC. Transferring T cells genetically redirected with anti-FRα-specific CARs represents an appealing strategy currently under investigation 13, 14. Anti-FRα-CAR-T cells have demonstrated effective killing activities against breast cancer cells *in vitro* 12. The tumor microenvironment (TME), including cellular components, extracellular matrix, cytokines, and chemokines, poses significant challenges to CAR-T cell therapy 15.

Cancer-associated fibroblasts (CAFs) are major components of the TME. They promote tumor growth 16 and induce programmed cell death ligand 1 expression in cancer cells 17. Fibroblast activation protein alpha (FAP) is a membrane-bound serine protease that mediates extracellular matrix remodelling 18. FAP is highly overexpressed in CAFs and has been identified as a promising target for CAF elimination 19. FAP-expressing CAFs exhibit pro-tumorigenic functions 20, 21 and create an immunosuppressive TME in breast cancer 22. Dendritic cells transfected with mRNA encoding FAP-lysosome-associated membrane protein-1 have been identified as candidate human stromal antigens. These cells can target and stimulate enhanced CD4^+^ and CD8^+^ T cell responses for breast cancer killing 19.

8-*O*-methylfusarubin (OMF) is a pyranonaphthoquinone natural product first isolated from the seagrass-derived fungus *Pestalotiopsis* sp. PSU-ES180 23, 24. It exhibits several biological activities, including anti-plasmodial, anti-malarial, and anti-tumor activity 25. OMF was selected as a CAF-targeting drug that may enhance stromal modulation and improve immunotherapy efficacy. However, specificity and poor intracellular uptake remain limitations of OMF 26. To address these issues, polylactic-co-glycolic acid (PLGA) NPs have been widely employed as drug delivery systems. PLGA NPs have received US Food and Drug Administration approval due to their biocompatibility and biodegradability 27. Our team recently developed anti-FAP-coated NPs containing OMF (anti-FAP@OMF-NPs). These NPs demonstrated enhanced toxicity against CAFs with high FAP expression compared with low-FAP CAFs from breast cancer tissue 28.

This study investigated whether anti-FAP@OMF-NPs could enhance the anti-tumor activity of anti-FRα-CAR-T cells. We evaluated this combination therapy using 3D co-cultured heterospheroids (HSs) containing high FRα-expressing TNBC cells and high FAP-expressing CAFs. The findings demonstrate potent cytotoxic effects from the combinatorial administration of anti-FRα-CAR-T cells, directed against breast cancer cells, and anti-FAP@OMF-NPs. Anti-FRα-CAR-T cells targeted breast cancer cells, whilst anti-FAP@OMF-NPs targeted CAFs. This dual approach effectively eliminated 3D cancer/CAF HSs that recapitulate the tumor environment of patient-derived tissues. These findings suggest significant therapeutic potential for this dual-targeting strategy in the effective eradication of solid tumor masses in clinical settings.

## Materials and Methods

### Blood collection

Peripheral blood mononuclear cells (PBMCs) were collected from 5 healthy donors (HDs) with written informed consent. The protocol was approved by the Siriraj Institutional Review Board, Faculty of Medicine Siriraj Hospital, Mahidol University (COA no. Si 182/2023).

### Cell lines and cell culture

Normal mammary epithelial MCF-10A cells (#CRL-10317, ATCC) were cultured in Dulbecco's Modified Eagle's Medium/F12 (DMEM/F12; Gibco). The medium was supplemented with 5% horse serum (Invitrogen), 100 U/ml penicillin (Invitrogen), 100 μg/ml streptomycin (Invitrogen), and 20 ng/ml epidermal growth factor (Peprotech). Additional supplements included 0.5 mg/ml hydrocortisone (Sigma-Aldrich), 100 ng/ml cholera toxin (Sigma-Aldrich), and 10 μg/ml insulin (Sigma-Aldrich). Human MDA-MB-231 cells (#HTB-26, ATCC) and human embryonic kidney cells (Lenti-X HEK293T, #632180, Takara Bio USA, Inc.) were co-cultured in DMEM (Gibco). This medium contained 10% fetal bovine serum (FBS), 100 U/ml penicillin, and 100 μg/ml streptomycin. Normal human dermal fibroblast alpha cells (HDFa, PCS-01-012, ATCC) and in-house established primary culture CAFs were also maintained. These CAFs (PC-B-004CAFs, PC-B-130CAFs, and PC-B-132CAFs) were isolated from breast cancer patients. The isolation protocol was approved by Siriraj Institutional Review Board (COA no. Si 329/2017). CAFs and HDFa cells were cultured in DMEM/F12 supplemented with 20% FBS, 100 U/ml penicillin, and 100 μg/ml streptomycin. All cells were maintained at 37°C in a 5% CO_2_ humidified incubator. All cell lines underwent routine mycoplasma contamination testing.

### Synthesis of OMF-loaded NPs and antibody functionalisation

FAP-targeted OMF-loaded NPs (anti-FAP@OMF-NPs) were synthesised using a standard emulsion solvent evaporation method previously described 28. OMF was dissolved in dimethyl sulphoxide and added dropwise to a PLGA solution. The first emulsion was performed by sonicating this mixture with an ultrasonic probe sonicator (Sonics and Materials Inc.). Sonication occurred at 30% amplitude in an ice bath. The mixture was then emulsified in 2% w/v polyvinyl alcohol. The NP solution was subsequently stirred at room temperature to remove the organic solvent. OMF-NPs were recovered using a Sorvall RC6+ centrifuge (Thermo Fisher Scientific) at 12000 rpm for 10 min. Prepared NPs were washed, resuspended in water, and followed by lyophilised. To conjugate human anti-FAP antibody (MAB3715, R&D System) to lyophilised NPs, the synthesised NPs were activated. The activation used carbodiimide and sulpho-NHS coupling reagents in 0.45 mM MES buffer at pH 4.7 for 2 h. Activated NPs were recovered by centrifugation and conjugated with anti-FAP antibody in sterile 1X phosphate-buffered saline (PBS) pH 7.4. The final antibody concentration was 8 μg/ml. Finally, the FAP-targeted OMF-loaded NPs were dissolved in sterile water for injection. The final product was stored at 4°C for subsequent experiments.

### Characterization of OMF-loaded NPs

The physicochemical characteristics of the synthesized NPs were comprehensively evaluated. Hydrodynamic diameter and zeta potential were analyzed using a Zetasizer Nano ZS (Malvern Instruments Ltd., Malvern). The encapsulation efficiency and loading capacity of OMF were quantified by ultraviolet-visible spectrophotometry (Synergy HT; BioTek Instruments Inc.) through absorbance measurement at 490 nm. Antibody conjugation efficiency was quantified by the Bradford protein assay.

### The stability and drug release of the OMF-loaded NPs

The *in vitro* release profile of OMF was assessed by incubating anti-FAP@OMF-NPs in PBS (pH 7.4) at 37°C under constant agitation. At predetermined time intervals, samples were collected and centrifuged at 12,000 rpm for 10 min using a Sorvall RC6+ centrifuge (Thermo Fisher Scientific). NP stability was examined by monitoring changes in hydrodynamic size and zeta potential using a Zetasizer Nano ZS during a 3-day incubation in complete medium at 37°C.

### Internalization of OMF-loaded NPs into CAFs

CAFs at 2 x 10⁴ cells/well were seeded on sterilized glass-bottom dishes and cultured for 24 h to reach 80% confluence. Cells were washed once with warm 1x PBS and incubated with NPs. Herein, coumarin-6 (Cou6) was used as a representative of OMF. The cells were incubated with Cou6@NPs or Cou6@NPs-anti-FAP working solutions at 1 mg/ml (high dose) or 100 µg/ml (low dose) in serum-free DMEM for 1 h at 37°C. Serum-free DMEM wells served as negative controls. After incubation, cells were washed with 1x PBS to remove unbound NPs. To quench any remaining surface-bound fluorescence, acquired using a confocal microscope (LSM800, Carl Zeiss Microscope). The acquisition software was ZEN 2.3 (Blue edition, 2002-2011).

### Characterisation of cancer cell-CAF HSs by immunohistochemistry staining

The phenotype of cancer/fibroblast spheroids was produced and characterised. To form 3D-HSs (co-cultured spheroids), cancer cells and CAFs were mixed at a 1:3 ratio. Cancer cells included MDA-MB-231 and MCF-10A cells. CAFs included HDFa, PC-B-004CAFs, PC-B-130CAFs, and PC-B-132CAFs. A total of 1×10³ cells per sphere were seeded in 100 μl DMEM. Cells were plated in 96-well clear round-bottom ultra-low attachment microplates (7007, ULA plate, Corning). Plates were centrifuged at 500 × *g* for 5 min at 4°C to induce cell aggregation. Spheroids were subsequently maintained at 37°C with 5% CO_2_ in a humidified incubator. After 48 h of incubation, spheroids were ready for experimental use.

Formalin-fixed paraffin-embedded (FFPE) spheroids underwent immunohistochemical staining. Primary antibodies included 1:200 anti-pan-cytokeratin (panCK) (sc-8018, Santa Cruz), 1:200 anti-α-smooth muscle actin (anti-α-SMA, A5228, Sigma-Aldrich), 1:100 anti-FAP (ab53066, Abcam), and 1:100 mouse anti-FRα antibody (MA5-23917, Thermo Fisher Scientific). After washing, EnVision+ System HRP anti-rabbit antibody (K4002, Dako, Agilent Technologies) was applied. Peroxidase activity was detected using diaminobenzidine (DAP). In addition, the FFPE spheroid slides were rinsed with distilled water before haematoxylin staining. Slides underwent dehydration in successive alcohol and xylene baths. Finally, slides were mounted with Pertex solution (Histolab).

### Double immunofluorescence staining

MCF-10A, MDA-MB-231, HDFa, PC-B-004CAFs, PC-B-130CAFs, and PC-B-132CAFs underwent double immunofluorescence staining. Cells were incubated with primary antibodies overnight at 4°C. These included 1:100 mouse anti-FRα antibody (MA5-23917, Thermo Fisher Scientific) and 1:100 rabbit anti-FAP antibody (ab53066, Abcam). After washing, cells were stained with secondary antibodies. The secondary antibodies comprised 1:200 goat anti-mouse IgG, Cy3 (115-166-071, Jackson ImmunoResearch Inc.), and 1:200 donkey anti-rabbit IgG, Alexa Fluor 488 (A21206, Life Technology). Nuclear staining was used 1:1000 Hoechst 33342 (H3570, Thermo Fisher Scientific) applied for 2 h at room temperature. Staining occurred in a light-protected environment. Cells were washed twice with 1X PBS before visualisation. Images were acquired using a confocal microscope (LSM800, Carl Zeiss Microscope). The acquisition software was ZEN 2.3 (Blue edition, 2002-2011).

### Flow cytometry evaluation of FRα and FAP

MCF-10A, MDA-MB-231, HDFa, PC-B-004CAFs, PC-B-130CAFs, and PC-B-132CAFs were evaluated for FRα and FAP expression. Cell pellets were blocked with 5% bovine serum albumin (BSA) in 1X PBS for 30 min. Cells were then stained with primary antibodies for 1 h at room temperature. The primary antibodies included 1:100 mouse anti-FRα antibody (MA5-23917, Thermo Fisher Scientific) and 1:100 rabbit anti-FAP (ab53066, Abcam). Following two washes, cells were incubated with secondary antibodies for 1 h at room temperature. The secondary antibodies comprised 1:200 goat anti-mouse IgG, PE (A28175, Thermo Fisher Scientific) and 1:200 donkey anti-rabbit IgG, Alexa Fluor 488 (A21206, Life Technology). Cells were examined using a BD Accuri C6 Plus Flow Cytometer (BD Bioscience). Data were analysed using FlowJo 10 software (Tree Star).

### Production of anti-FRα-CAR-T cells

#### Plasmid construction

The 4^th^-generation anti-FRα-CAR constructs were kindly provided by Dr. Piriya Luangwattananun, the Division of Molecular Medicine and Siriraj Center of Research Excellence for Cancer Immunotherapy, Research Department, Faculty of Medicine Siriraj Hospital, Mahidol University 12. The FRα-CAR construct contains anti-FRα-specific single-chain variable fragment (scFv) derived from MORAb-003 antibody. This scFv is linked to CD28, 4-1BB, and CD27 co-stimulatory domains and the CD3ζ cytosolic domain 12.

#### Lentiviral transduction

HEK293T cells were transfected with pMD2.G, psPAX2, and pCDH-FRα-CAR4 plasmids using the calcium phosphate-mediated transfection method 12. Transfected HEK293T cells were cultured in DMEM supplemented with 10% FBS. Anti-FRα-CAR-carrying lentivirus-containing supernatants were collected at 48 h and 72 h. Collected supernatants were stored at 4°C. Lentivirus-containing supernatants underwent concentration by high-speed centrifugation at 20000 × *g* for 90 min at 4°C. Viral titre was quantified using a qPCR Lentiviral Titration Kit (LV900, Applied Biological Materials) according to the manufacturer's protocol. Activated T cells were transduced with FRα-CAR lentiviruses at a multiplicity of infection (MOI) of 50. Transduction used 10 μg/ml protamine sulphate and spinoculation at 1200 × *g* for 90 min at 32°C. The plate was then incubated for 24 h. Transduced T cells were maintained in AIM-V medium (Gibco) supplemented with 5% human AB serum (H4522, Sigma-Aldrich). The medium contained 20 ng/ml rhIL-2 (11340025, ImmunoTools), 10 ng/ml rhIL-7 (11340075, ImmunoTools), and 40 ng/ml rhIL-15 (11340155, ImmunoTools). Cells were cultured for 2 days before analysis of surface FRα-CAR expression and functional assays.

### Characterisation of CAR transduction

The gating strategy for anti-FRα-CAR-T cells used pseudo-colour plots of FSC-H (size) and SSC-H (granularity) on a linear scale. Initial gating identified the lymphocyte population. Because non-lymphocyte cells were removed during CAR-T cell production, plots showed only 2 populations: cell debris and targeted lymphocytes. Single cells were then gated using FSC-H and FSC-A parameters. Subsequently, T cells were identified using 1:200 Brilliant Violet 421 anti-CD3 antibody (300433, BioLegend). Anti-FRα-CAR-T cells were gated. Then, the positive expression of the anti-Myc tag Ab (ab1394, Abcam) staining on CD3^+^ T cells was considered as anti-FRα-CAR-T cells. Moreover, the subpopulation of CAR-T cells was gated using 1:200 AF700 anti-CD4 Ab (317425, BioLegend), PerCP anti-CD8 Ab (21810085, ImmunoTools), APC anti-CD45RA Ab (21819456, ImmunoTools), and PE anti-CD62L Ab (21819624, ImmunoTools). The cells were acquired by CytoFLEX-S (Beckman Coulter). Data were analysed using FlowJo 10 software (Tree Star).

### Preparation of peripheral blood mononuclear cells (PBMCs) and primary T cell activation

PBMCs were collected from 5 healthy donors' blood and isolated using Lymphocyte Separation Medium (Corning) according to the manufacturer's protocol. Twenty ml of blood from each healthy donor was collected and diluted with an equal volume of PBS (Gibco). Diluted blood was overlaid on Lymphocyte Separating Medium (Corning) and centrifuged at 800 × *g* for 30 min at room temperature. The buffy coat layer containing PBMCs was collected and transferred to a new centrifuge tube. PBMCs were washed twice with PBS. Obtained PBMCs were cultured in AIM-V medium (Sigma-Aldrich) for 24 h. The non-adherent cells containing T cells were collected. These cells were activated using 5 μg/ml phytohemagglutinin-L (PHA-L, 11249738001, Roche) in AIM-V medium. The medium was supplemented with 20 ng/ml IL-2 (ImmunoTools GmbH) for 72 h.

### T cell subset analysis using flow cytometry

T cell phenotypes of CAR-T cells were characterised using specific antibodies. These included 1:200 Brilliant Violet 421 anti-human CD3 antibody (300433, BioLegend), 1:200 Alexa Fluor 700 anti-human CD4 antibody (317425, BioLegend), and 1:200 PerCP anti-human CD8 antibody (21810085, ImmunoTools). Cells were acquired using CytoFLEX-S. Data were analysed using FlowJo 10 software.

### Cancer cell killing by 3D co-cultured breast cancer cells and CAF heterospheroids (HSs)

Three-dimensional homospheroids (HOs) and HSs were generated to mimic the stromal-rich environment in breast cancer patient tissue. To distinguish different cell populations in co-culture spheroids, cells were pre-stained with fluorescent dyes. MDA-MB-231 TNBC cells and normal epithelial mammary MCF-10A cells were pre-stained with CellTracker Green CMFDA Dye (C2925, Invitrogen). Normal HDFa and in-house established primary CAFs (PC-B-004CAFs, PC-B-130CAFs, and PC-B-132CAFs) were pre-stained with CellTracker Orange CMRA Dye (C34551, Invitrogen). Primary CAFs included PC-B-004CAFs, PC-B-130CAFs, and PC-B-132CAFs. Three-dimensional spheroids were generated using 96-well clear round-bottom ultra-low attachment microplates (ULA plate, Corning) with 2.5% Matrigel (354234, Corning). Each 3D-HO comprised 1×10³ cells in 100 μl DMEM. Cell types included MCF-10A, MDA-MB-231, HDFa, PC-B-004CAFs, PC-B-130CAFs, and PC-B-132CAFs. To form co-cultured 3D-HSs, cancer cells (MDA-MB-231 and MCF-10A cells) and CAFs (HDFa, PC-B-004CAFs, PC-B-130CAFs, and PC-B-132CAFs) and CAFs were mixed at a 1:3 ratio. This totalled 1×10³ cells per microsphere in 100 μl DMEM. Cancer cells included MDA-MB-231 and MCF-10A cells. CAFs included HDFa, PC-B-004CAF, PC-B-130CAF, and PC-B-132CAF. Plates were centrifuged at 500 × *g* for 5 min at 4°C to induce cell aggregation. Spheroids were subsequently maintained at 37°C with 5% CO_2_ in a humidified incubator. After 48 h of incubation, spheroids were ready for experimental use.

Fluorescence signals from 3D-HOs and 3D-HSs were detected using inverted fluorescence microscopy. Images were acquired via the CellSense Standard program version 1.15 (Olympus). Spheroids underwent treatment under various conditions for 2 days. Treatment conditions: 1) target alone (no treatment), 2) mock T cells (effector to target ratio (E:T) = 20:1), 3) anti-FRα-CAR-T cells (E:T = 20:1), 4) anti-FAP@OMF-NPs (1 mg/ml), and 5) anti-FRα-CAR-T cells (E:T = 20:1) + anti-FAP@OMF-NPs (1 mg/ml) for 2 days. The fluorescence intensity (FI) of spheroids was measured using ImageJ software (National Institutes of Health, USA, https://imagej.nih.gov/ij/). The percentage of cell lysis of targeted cells was calculated using the formula as shown below.



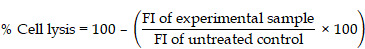



### Multiplex cytokine bead array

Secreted cytokine production from CAR-T cells was measured using LEGENDplex Human CD8/NK Panel (Cytokine Bead Array, CBA; 741065, BioLegend). The assay followed the manufacturer's protocol. This CBA detects 13 human cytokines: IL-2, IL-4, IL-6, IL-10, IL-17A, IFN-γ, TNF-α, sFas, sFasL, granzyme A, granzyme B, perforin, and granulysin. Cytokine levels were measured using a CytoFLEX flow cytometer. Data were analysed using LEGENDplex Data Analysis Software Suite (BioLegend).

### Statistical analysis

All experiments were conducted with at least two independent replicates. Results were reported as means with standard deviations. Statistical analyses were performed using Student's *t* test and ANOVA. Analyses used GraphPad Prism version 10 (GraphPad Software). A *p* value < 0.05 was considered statistically significant.

## Results

### Surface FRα and FAP expression in breast cancer cells and primary culture CAFs

**Fig 1:**
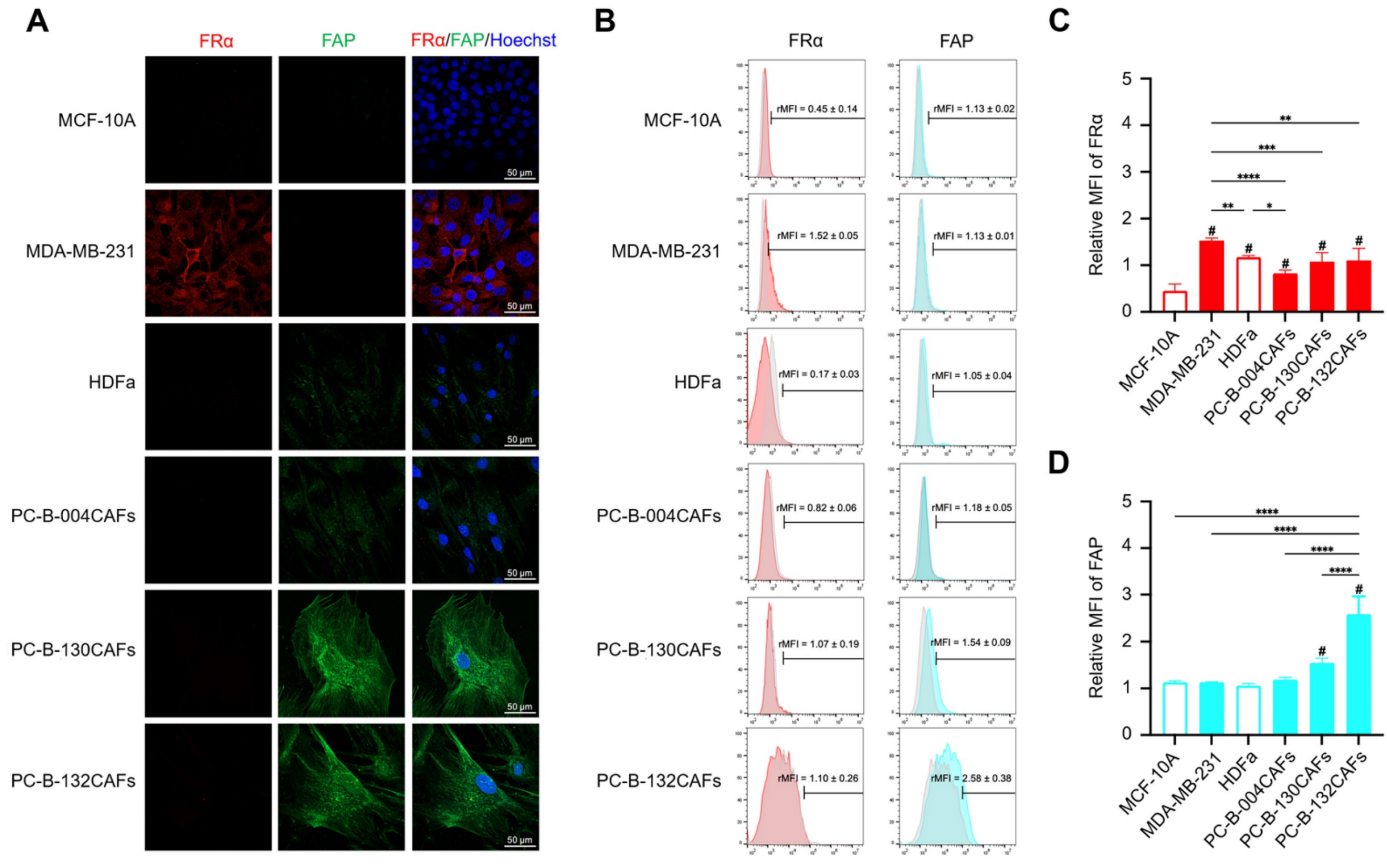
** The expressions of FR⍺ and FAP in breast epithelial cells and fibroblasts. (A)** Immunofluorescence staining showing FRα (green) and FAP (red) expression in MCF-10A, MDA-MB-231, HDFa, PC-B-004CAFs, PC-B-130CAFs, and PC-B-132CAFs. **(B)** Flow cytometry analysis of FRα and FAP expression. **(C)** Relative mean fluorescence intensity (MFI) of FRα expression (# indicates *p* < 0.05 compared to MCF-10A) and **(D)** Relative MFI of FAP expression (# indicates *p* < 0.05 compared to HDFa). The pictures indicated the replicates of three independent experiments; *p < 0.05; **p < 0.01, ***p < 0.001, and ****p < 0.0001. Original magnification = 200x; scale bar is 50 μm.

FRα expression (red fluorescence color) was prominently detected in MDA-MB-231 cells compared with MCF-10A cells and fibroblasts. FAP expression (green fluorescence color) was higher in PC-B-130CAFs and PC-B-132CAFs than in PC-B-004CAFs and normal fibroblasts (**Fig. 1A**). Flow cytometry results demonstrated high FRα expression in MDA-MB-231 cells. The relative mean fluorescence intensity (rMFI) was 1.526 ± 0.059 in MDA-MB-231 while PC-B-130CAFs and PC-B-132CAFs exhibited high FAP levels, with rMFI values of 1.548 ± 0.010 and 2.580 ± 0.384, respectively (**Fig. 1B and 1D**). In contrast, both MCF-10A and HDFa showed low levels of FRα and FAP on their surfaces (**Fig. 1C and 1D**). The highest FRα expression occurred in MDA-MB-231 cells, whilst the highest FAP expression occurred in PC-B-132CAFs. These results indicate specific and predominant expression of FRα in TNBC cells and FAP in certain CAFs (**Fig. 1C and 1D**).

### Characterisation of 3D cancer cell-CAF heterospheroids

**Fig 2:**
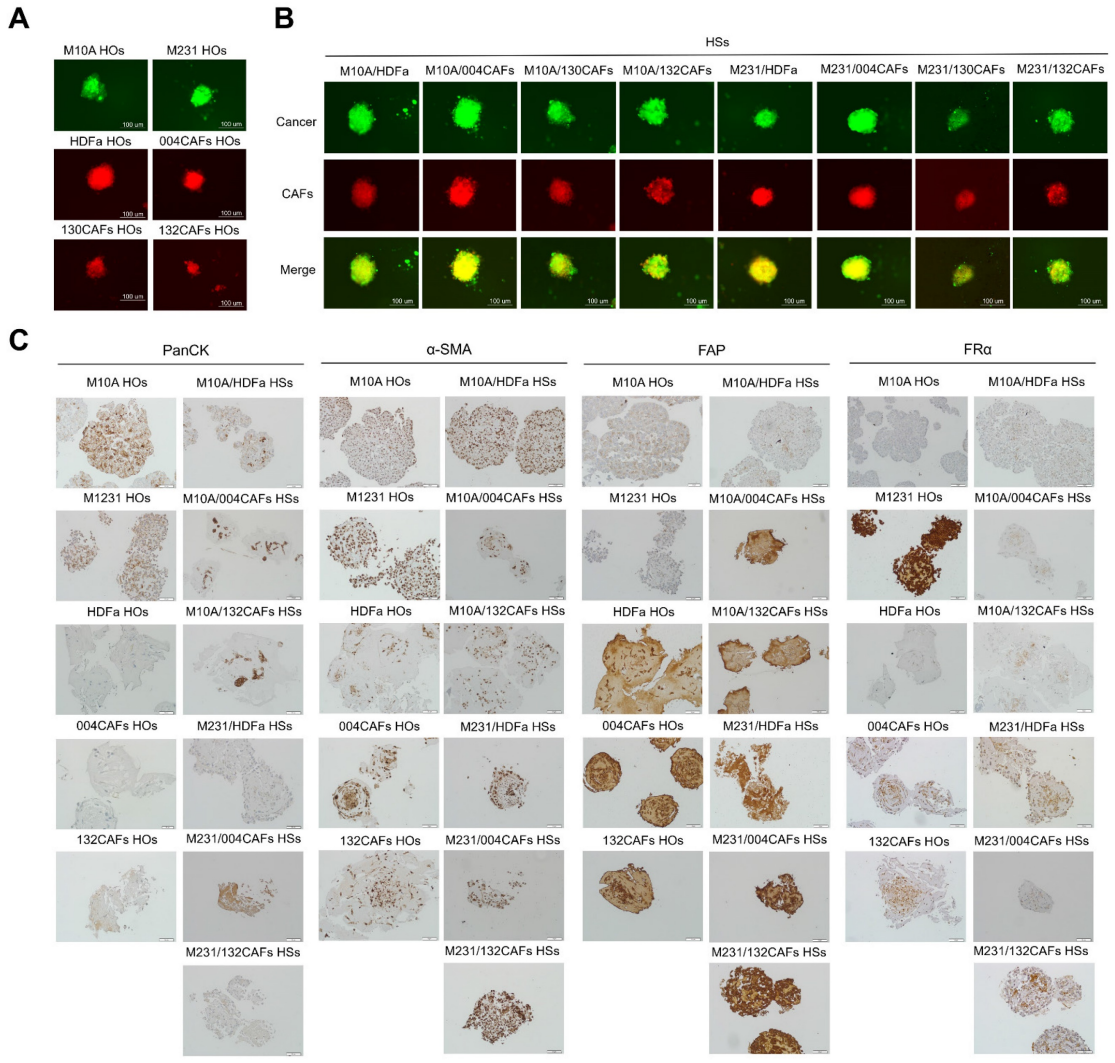
**Immunofluorescence and immunohistochemistry staining of panCK, ⍺-SMA, FR⍺, and FAP in HOs and HSs. (A)** Fluorescence images of HOs (M10A, M231, HDFa, 004CAFs, 130CAFs, and 132CAFs) and **(B)** HSs (M10A/HDFa, M10A/004CAFs, M10A/130CAFs, M10A/132CAFs, 231/HDFa, M231/004CAFs, M231/130CAFs, and M231/132CAFs) using CellTracker CMFDA (green color = cancer) and CMRA (red color = CAFs). Original magnification = 630x; scale bar is 100 μm. **(C)** panCK, α-SMA, FR⍺, and FAP expressions by immunohistochemistry of HOs and HSs. Original magnification = 200x; scale bar is 50 μm.

Co-cultured 3D-HSs were successfully generated using breast epithelial cells mixed with fibroblasts. Either MCF-10A normal breast cells (M10A) or MDA-MB-231 breast cancer cells (M231) were combined with 4 distinct fibroblasts including HDFa, PC-B-004CAFs (004CAFs), PC-B-130CAFs (130CAFs), and PC-B-132CAFs (132CAFs) at a 1:3 ratio. Each spheroid contained 1000 cells total. HOs of individual cell types were also produced using 1000 cells per spheroid. Green fluorescence signal represents viable breast cancer cells whilst red fluorescence signal represents viable fibroblasts. Representative images of 3D-HOs and 3D-HSs are shown in **Fig. 2A and 2B**. All 3D-HOs and 3D-HSs displayed round shapes with complex cancer structures. Cancer cells (green signal) infiltrated and concentrated in the centre of 3D-HSs (**Fig. 2B**).

Immunohistochemistry staining was performed using anti-pan-cytokeratin (panCK), anti-α-SMA, anti-FRα, and anti-FAP antibodies of HOs and HSs revealed the positive signal of panCK phenotypes in all M10A HOs and M231 HOs, and the center of M10A/HDFa HSs, M10A/004CAFs HSs, M10A/132CAFs HSs, M231/HDFa HSs, M231/004CAFs HSs, and M231/132CAFs HSs (**Fig. 2C**). The α-SMA staining, indicating activated fibroblast markers, was detected in both cancerous and CAF cluster balls. Strong α-SMA signals appeared in M231/132CAF HSs. Similarly, FAP was strongly detected in 132CAF HOs and M231/132CAF HSs. However, FAP expression also occurred in MDA-MB-231 breast cancer cells and normal fibroblast as previously reported 29. Furthermore, FRα was highly expressed in M231 HOs more than that of M10A HOs. Interestingly, CAFs showed a low level of FRα supported by some previous work 30. All 3 types of HSs containing MDA-MB-231 breast cancer cells (M231/HDFa HSs, M231/004CAFs HSs, and M231/132CAFs HSs) had high FRα presenting on cancer cells (**Fig. 2C**).

### Anti-FRα-CAR-T cells were successfully generated and CD8+ T cells were predominant

**Fig 3:**
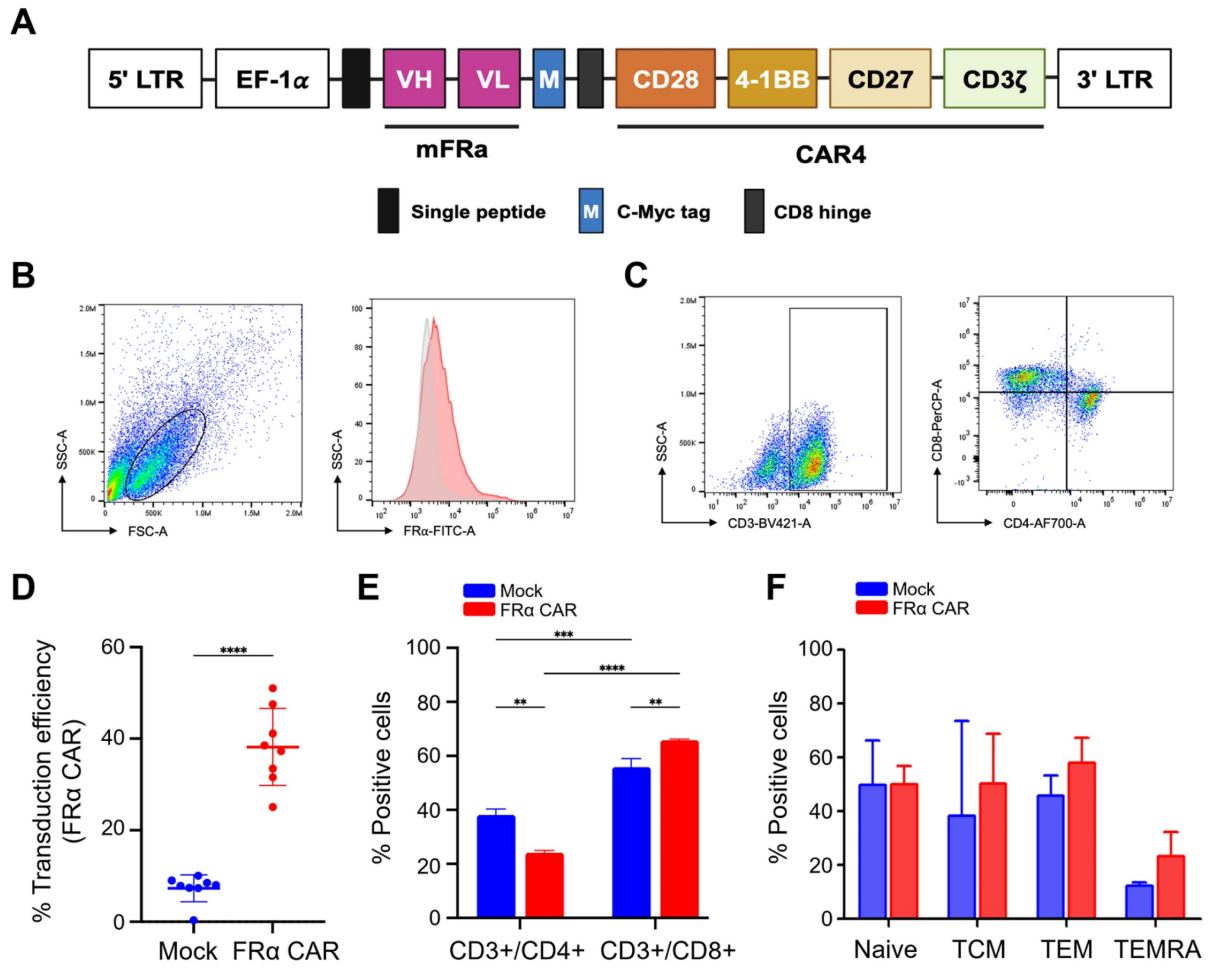
**Characterisation of FRα-CAR-T cells. (A)** Construction of FR⍺-CAR containing EF-1⍺ promotor, anti-FR⍺ mAb-derived scFv, multiple co-stimulatory molecules (CD28, 4-1BB, and CD27), and CD3ζ. **(B)** Gating strategy for FR⍺-CAR-T cell population and **(C)** CD3^+^/CD4^+^ helper T cell and CD3+/CD8+ cytotoxic T cell. **(D)** Percentage of transduction efficiency of FR⍺-CAR-T cell and mock T (MT) cell summarized from independent experiments from five HDs. **(E)** An average percentage of helper T cells (CD3^+^/CD4^+^) and cytotoxic T cells (CD3^+^/CD8^+^) in anti-FRα-CAR-T cells and MT was summarized from two independent experiments. **(F)** Percentage of the T cell subset analysed with anti-CD3, anti-CD45RA, and anti-CD62L antibodies for anti-FRα-CAR-T cell and mock T cell summarized from three independent experiments. **p < 0.01, ***p < 0.001, and ****p < 0.0001.

The CAR construct contained anti-FRα-specific scFv derived from MORAb-003 antibody. This scFv was linked to CD28, 4-1BB, and CD27 co-stimulatory domains and the CD3ζ cytosolic domain (**Fig. 3A**). Following activation and lentiviral transduction, anti-FRα-CAR-T cells were analysed using the gating strategy shown in **Fig. 3B**. Anti-FRα-CAR-T cells exhibited 37.61% ± 7.310% anti-FRα-CAR protein expression on the cell surface. This compared with 7.801% ± 2.610% in mock T cells (**Fig. 3D**). Representative dot plots confirmed surface expression of FRα-CAR predominantly on CD3⁺ T cells, with both CD4⁺ or CD8⁺ expressing CAR **(Fig. 3C).** T cell subset analysis revealed markedly higher CD8^+^ cytotoxic T cells (65.750% ± 0.495%) than CD4^+^ helper T cells (24.150% ± 0.919%; **Fig. 3E**). CAR gene transduction significantly activated CD8^+^ cytotoxic T cells compared with mock T cells (**Fig. 3E**).

T cell subsets were evaluated, including naive T cells (CD3^+^/CD45RA^+^/CD62L^+^), central memory T cells (TCM; CD3^+^/CD45RA^-^/CD62L^+^), effector memory T cells (TEM; CD3^+^/CD45RA^-^/CD62L^-^) and terminal effector T cells (TEMRA; CD3^+^/CD45RA^+^/CD62L^-^) were evaluated (**Fig. 3F**). Phenotypic analysis of T-cell subsets showed no significant difference was observed among mock T cells and anti-FRα-CAR-T cells, however, the TEM and TEMRA phenotypes were the majorities of HD01's and HD02's anti-FRα-CAR-T cells which were increased when compared to mock T cells (**Fig. 3F**).

### Increased anti-tumor activity of combined FRα-CAR-T cells and 8-*O*-methylfusarubin-(OMF)-encapsulated anti-FAP-coated NPs

We first synthesized and characterized the OMF-encapsulated anti-FAP-coated NPs (anti-FAP@OMF-NPs) 28. As shown in the **Supplementary Table S1**, the synthesized OMF-NPs exhibited a hydrodynamic size of 201.33 ± 3.63 nm and a zeta potential of -23.30 ± 2.68 mV. Upon antibody conjugation, the hydrodynamic size slightly increased to 215.17 ± 2.61 nm, and the surface charge shifted toward a less negative value (-14.22 ± 0.49 mV), indicating successful antibody attachment. The encapsulation efficiency of OMF was determined to be 72.68 ± 1.64%, comparable to the previously reported value 31, and the OMF loading capacity was 8.26 ± 0.19%, which aligns with other studies utilizing PLGA as a carrier (10 ± 1.2%) 31. Furthermore, the antibody conjugation efficiency was 58.74 ± 3.94%, which is consistent with the previous report (43.78 ± 14.18%) 28. Collectively, these results confirm that the NPs closely reproduce the physicochemical characteristics of the original formulation, demonstrating the robust reproducibility and reliability of our nanosystem for further biological studies.

In addition, anti-FAP@OMF-NPs exhibited excellent stability in cell culture medium containing 10% FBS over a 3-day period, with no significant changes in hydrodynamic size or zeta potential, indicating good structural integrity **(Supplementary Fig. S1A)**. Cell culture medium supplemented with 10% FBS provides a physiologically relevant *in vitro* environment to evaluate NP stability, enabling assessment of serum protein interactions and protein corona formation 32-34. Consistent with previous reports on PLGA-based nanosystems, including BSA-PLGA NPs, the formulation exhibited excellent colloidal stability in both PBS and FBS-containing media for up to 72 h, with minimal changes in hydrodynamic size and surface charge 35, 36. Together with our previous demonstration of PLGA NP hemocompatibility, including low hemolysis and no coagulation effects 37, this high colloidal stability supports the suitability of anti-FAP@OMF-NPs for systemic application. Moreover, the drug release profile of anti-FAP@OMF-NPs was examined and displayed a biphasic pattern (**Supplementary Fig. S1B**), characterized by an initial burst release of 65.58 ± 5.73% within 8 h, followed by a sustained release phase reaching 96.92 ± 5.48% at 72 h. This controlled and prolonged release behavior, together with the high colloidal stability, highlights the potential of this nanosystem to provide efficient and sustained drug delivery.

We next evaluated the NPs' internalization efficiency in both FAP-high and FAP-low CAFs models. Confocal imaging analyses revealed that 1 mg/ml and 100 μg/ml of Cou6@NPs or Cou6@NPs-anti-FAP were preferentially internalized into their respective FAP-high-expressing target cells, including PC-B-120CAFs, PC-B-130CAFs, and PC-B-132CAFs, whereas minimal uptake was observed in low-expressing counterparts (PC-B-140CAFs and PC-B-142CAFs), indicating limited NP uptake **(Supplementary Fig. S2)**. These results further confirm the target specificity of the NPs and support their selective activity toward FAP-positive tumor subpopulations.

The anti-tumor activity of a single treatment of CAR-T cells or CAFs-targeting NPs and their combination on HSs or HOs was exerted by fluorescence imaging. Treatments included CAR-T cells alone, CAF-targeting NPs alone, and their combination on either HOs or HSs. Green fluorescence signals represented viable cancer cells, whilst red signals represented viable fibroblasts. Anti-FRα-CAR-T cells showed no toxicity to M10A HOs, however, these CAR-T cells significantly destroyed M231 HOs (**Supplementary Fig. S3A and S3B**). The anti-FAP@OMF-NPs had a low toxic effect on HDFa normal fibroblast and 004CAFs HOs, compared to high cell lysis detected in 130CAFs HOs and 132CAFs HOs (**Supplementary Fig. S3C and S34D**). Moreover, anti-FAP@OMF-NPs showed significantly increased killing activity on M231 HOs compared to the no treatment condition (**Supplementary Fig. S4A and S4B**).

The specificity of anti-FRα-CAR-T cells was observed in M231 HOs compared to mock T cells and target alone, while no significant normal MCF-10A spheroid killing by these CAR-T cells compared to that of mock T cells (**Supplementary Fig. S4A and S4B**). For CAFs with different FAP levels, the specific killing of anti-FAP@OMF-NPs was observed in high FAP-130CAFs HOs and high FAP-132CAFs HOs compared to low FAP-004CAFs HOs (**Supplementary Fig. S4C and S4D**).

**Fig 4:**
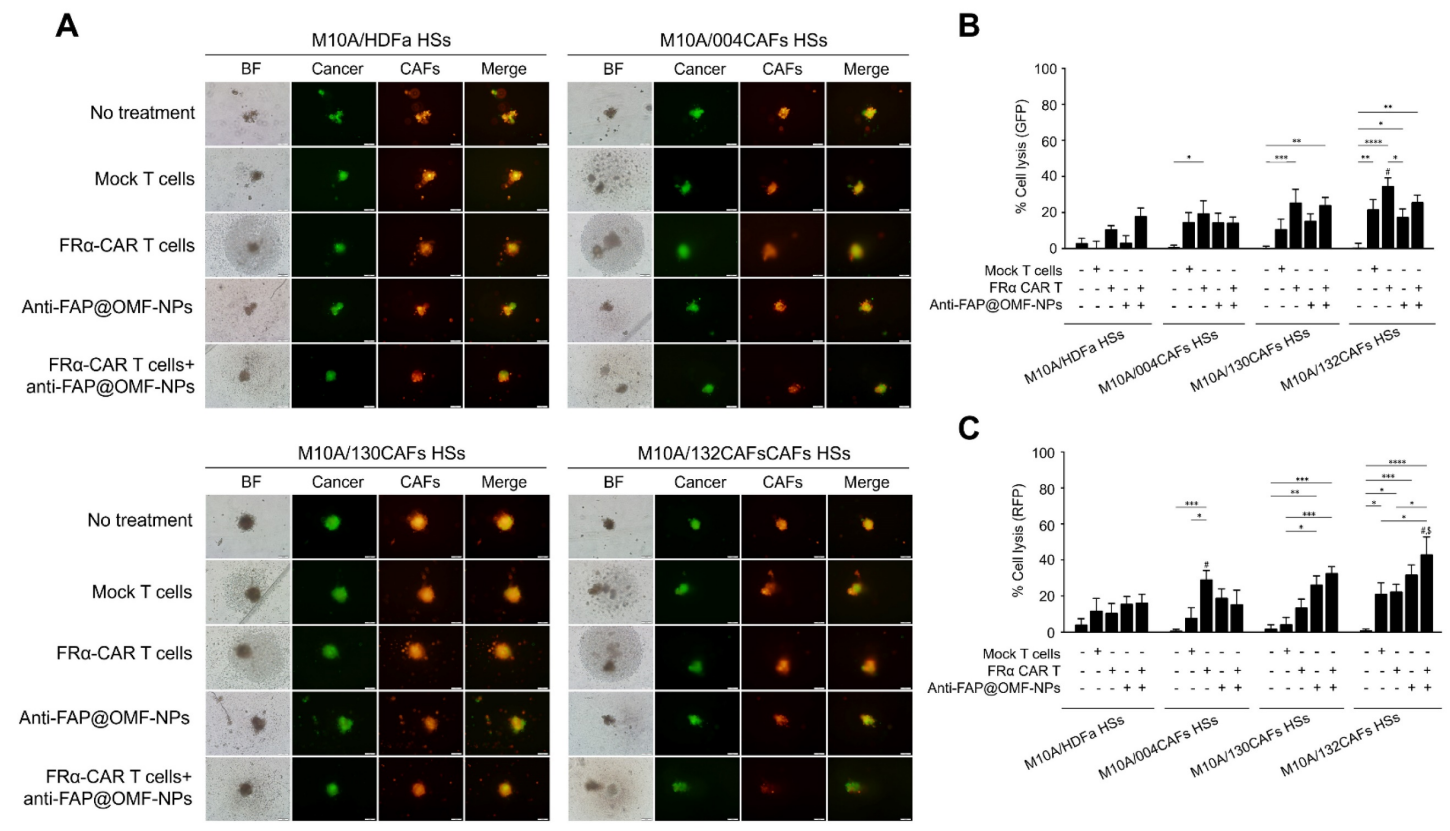
**The representative images and histogram of the % cell lysis of anti-FRα-CAR-T cells (produced from pooled results from HD01 to HD 05) and anti-FAP@OMF-NPs in 3D M10A/HDFa HSs, M10A/004CAFs HSs, M10A/130CAFs HSs, and M10A/132CAFs HSs. (A)** Fluorescence images (green color = cancer and red = CAFs) of 3D M10A/HDFa HSs, M10A/004CAFs HSs, M10A/130CAFs HSs, and M10A/132CAFs HSs after being treated with various treatment conditions for 48 h. **(B)** Percentage of cell lysis (green = cancer) of different spheroids at 48 h post-treatment (# indicates *p* < 0.05 compared to M10A/HDFa HSs). **(C)** Percentage of cell lysis (red = CAFs) of different spheroids at 48 h post-treatment (# indicates *p* < 0.05 compared to M10A/HDFa HSs and $ indicates *p* < 0.05 compared to M10A/004CAFs HSs). The results are summarized from five independent experiments (one experiment for one HD, in a total of 5 donors).

**Fig 5:**
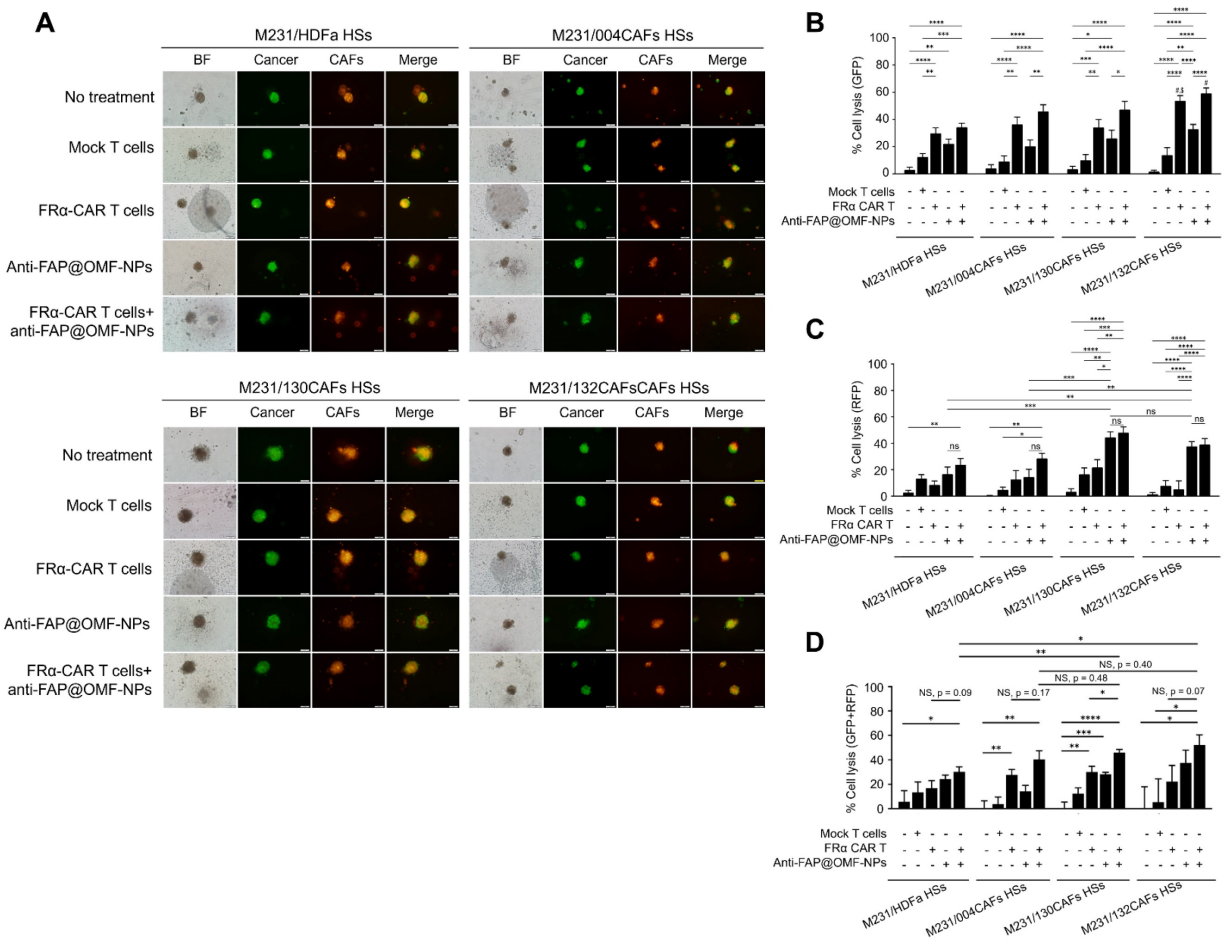
** The representative images and histogram of the % cell lysis of anti-FRα-CAR-T cells (produced from pooled results from HD01 to HD 05) and anti-FAP@OMF-NPs. (A)** Fluorescence images (green color = cancer and red = CAFs) of 3D M231/HDFa HSs, M231/004CAFs HSs, M231/130CAFs HSs, and M231/132CAFs HSs after being treated with various treatment conditions for 48 h. **(B)** Percentage of cell lysis (green = cancer) of different spheroids at 48 h post-treatment (# indicates p < 0.05 compared to M231A/HDFa HSs and $ indicates p < 0.05 compared to M231/004CAFs HSs). **(C)** Percentage of cell lysis (red = CAFs) of different spheroids at 48 h post-treatment. **(D)** Percentage of cell lysis (green + red signal) of 3D M231/HDFa HSs, M231/004CAFs HSs, M231/130CAFs HSs, and M231/132CAFs HSs. The results are summarized from five independent experiments (one experiment for one HD, in a total of 5 donors). NS = not significance. *p < 0.05, ***p < 0.01, ***p < 0.001, and ****p < 0.0001. The scale bar is 100 μm.

Results from the combined 5 HDs demonstrated the lowest cell lysis in MCF-10A cells (**Fig. 4A-C**). In contrast, the highest lysis occurred in MDA-MB-231 cells (**Fig. 5A-D**) after combined treatment. Combined anti-FRα-CAR-T cells and anti-FAP@OMF-NPs showed greater efficacy than either treatment alone. Individual signals were analysed separately: green fluorescent protein (GFP) for cancer cell lysis percentage and red fluorescent protein (RFP) for CAF lysis.

The combined anti-FRα-CAR-T cells and anti-FAP@OMF-NPs showed significantly higher killing activity in several conditions. First, enhanced killing occurred in M10A/132CAF HSs compared with M10A/HDFa HSs and M10A/004CAF HSs. This combination also exceeded anti-FRα-CAR-T cells alone (**Fig. 4C**; RFP signal). Second, increased killing was observed in M231/132CAF HSs compared with M10A/HDFa HSs and anti-FAP@OMF-NPs alone (**Fig. 5B**; GFP signal). In all HSs containing MDA-MB-231 cells, the combination treatment killed CAFs more effectively than treatment with anti-FAP@OMF-NPs alone (**Fig. 5C**; RFP signal).

This killing effect was analysed using combined fluorescence signals. GFP represented viable cancer cells, whilst RFP represented viable CAFs (**Fig. 5D**). Combined treatment with anti-FRα-CAR-T cells and anti-FAP@OMF-NPs significantly reduced both GFP and RFP signals. This reduction occurred in M231/130CAF HSs, indicating spheroid killing of these spheroids (**Fig. 5D**; **Supplementary Fig. S5**; **Supplementary Movie S1 and S2**) and M231/132CAFs HSs (**Fig. 5D**; **Supplementary Fig. S5**; **Supplementary Movie S1 and S3**) compared to M231/HDFa HSs (**Fig. 4**; **Supplementary Fig. S5**; **Supplementary Movie S1 and S4**) or to M231/004CAFs HSs after treatment (**Fig. 4**; **Supplementary Fig. S5**; **Supplementary Movie S5**).

### Secreted cytokines affect cancer cell-killing capability

**Fig 6:**
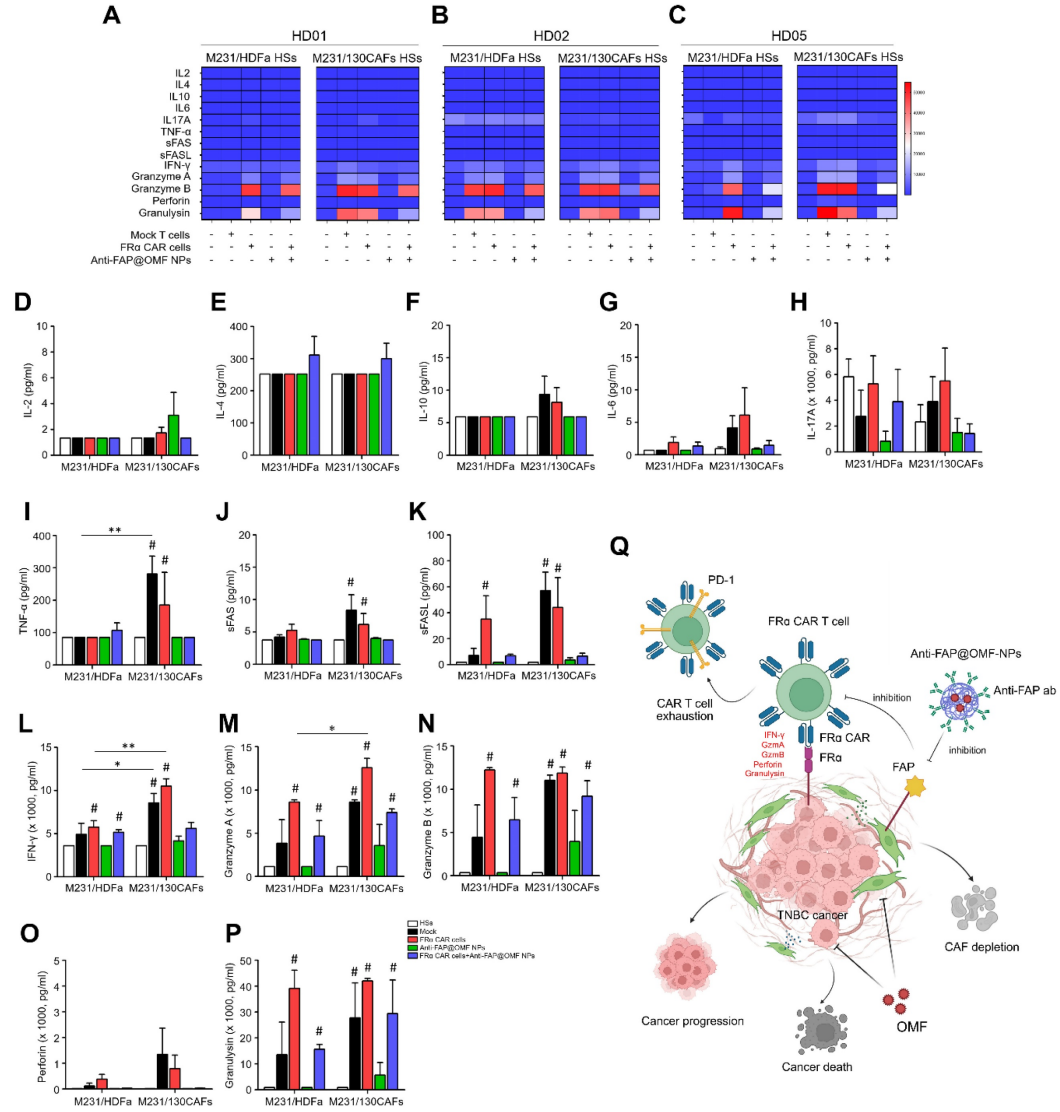
** Measurements of secreted cytokines in (A)** HD01, **(B)** HD02, and **(C)** HD05 including **(D)** IL-2, **(E)** IL-4, **(F)** IL-10, **(G)** IL-6, **(H)** IL-17A, **(I)** TNF-⍺, **(J)** sFas, **(K)** sFasL, **(L)** IFN-γ, **(M)** granzyme A, **(N)** granzyme B, **(O)** perforin, and **(P)** granulysin from different treatment conditions of heterospheroids (HSs as target cells), mock T cell, FR⍺-CAR-T cell, anti-FAP@OMF-NPs, and FR⍺-CAR-T cell + anti-FAP@OMF-NPs) after exposure to M231/HDFa and M231/130CAFs HSs. The result represents one independent experiment for each donor. **(Q)** Proposed mechanism of the study. Created with BioRender.com. # *p* < 0.05 compared to HSs, *p < 0.05, and ***p < 0.01.

Secreted cytokines were collected from M231/HDFa HSs and M231/130CAF HSs after treatment. Treatments included mock T cells, anti-FRα-CAR-T cells, anti-FAP@OMF-NPs, and combined treatment. TNF-α, sFAS, sFASL, IFN-γ, granzyme A, granzyme B, and granulysin levels were higher in anti-FRα-CAR-T cell-treated M231/130CAF HSs than in anti-FRα-CAR-T cell-treated M231/HDFa HSs (**Fig. 6A-P**). IFN-γ and granzyme A were significantly increased in M231/130CAF HSs exposed to anti-FRα-CAR-T cells compared with M231/HDFa HSs in each HD (**Fig. 6L and 6M**).

The obvious increase of granzyme A, granzyme B, and granulysin showed modest increases in both HS types. These increases occurred in M231/HDFa HSs and M231/130CAF HSs after combined CAR-T cell and NP treatment. Levels were elevated compared with untreated HSs (**Fig. 6M, N and P**). In contrast, elevated granzyme B and granulysin levels were observed only in M231/HDFa HSs. This elevation occurred with anti-FRα-CAR-T cell treatment and combined treatment in all HDs except HD02 (**Fig. 6B**). The increased granzyme B and granulysin from HSs treated with mock T cells compared to untreated HSs was observed from HD02 (**Fig. 6B**).

## Discussion

TNBC represents a distinct subtype within breast malignancies, distinguished by the absence of ER, PR, and HER2. TNBC accounts for approximately 15% - 20% of all breast cancer cases. It is associated with aggressive tumor behaviour, high recurrence rates, and poorer prognosis compared with other breast cancer subtypes 5. The TME frequently exhibits immunosuppressive features marked by diverse mechanisms. These mechanisms hinder CAR-T cell functionality and effectiveness 38. Immunomodulatory agents are released by CAFs, cancer cells, and immune cells. These include transforming growth factor beta (TGF-β), interleukin-10 (IL-10), and programmed cell death ligand 1 (PD-L1). Such agents can impede CAR-T cell infiltration, persistence, and anti-tumor responses 39, 40. Several studies suggest CAFs play crucial roles in promoting TNBC progression, invasion, metastasis, and therapy resistance 41. These factors could limit anti-FRα-CAR-T cell therapy effectiveness in overcoming TME immunosuppression 42.

The present study aimed to demonstrate enhanced anti-tumor activity through combined therapeutic approaches. We evaluated anti-FRα-CAR-T cells and anti-FAP@OMF-NPs against 3D co-cultured TNBC/CAF HSs. These HSs mimicked the stromal-rich environment found in breast cancer patients. FRα expression is variable across TNBC cell lines and patient tumors, which is preferentially expressed in a subset of basal-like TNBCs, while being low or absent in others 43. Specifically, FRα expression has been reported in commonly used TNBC cell lines such as MDA-MB-231, MDA-MB-436, MDA-MB-468, Hs578T, SUM159PT, and CAL-51, whereas moderate to low expression is observed in other TNBC models, including MDA-MB-453 and BT-549 44, 45. Based on this established expression pattern, MDA-MB-231 cells were selected as a representative FRα-positive TNBC model in the present study. High FRα-expressing MDA-MB-231 cells and high FAP-expressing CAFs were cultured as 3D-HSs to test the tumor. These models tested tumor killing capability of anti-FRα-CAR-T cells alone, anti-FAP@OMF-NPs alone, and combined treatment. The selectivity arises primarily through the anti-FAP antibody functionalization, which enables preferential binding and cellular uptake of OMF-loaded NPs by FAP-overexpressing CAFs. Following internalization, OMF exerts cytotoxic activity mainly via mitochondrial stress and reactive oxygen species (ROS)-mediated apoptosis 25. Treatment with OMF resulted in a significant increase in caspase-3/7 activity, leading to apoptotic cell death in CAFs 28. Additionally, based on structural similarities to other related fusarubin-type quinones, OMF may also engaged the extrinsic apoptotic pathway, as fusarubin has been shown to activate caspase-8 and upregulate FasL in cancer cells 28. Double immunofluorescence staining of FRα and FAP confirmed expression patterns alongside flow cytometry. CD8^+^ T cells predominated in anti-FRα-CAR-T cell production. These cells released several cytolytic enzymes upon exposure to target cancer spheroids. Moreover, anti-FAP@OMF-NPs revealed increased killing of FAP-positive cells. Combined treatment with anti-FRα-CAR-T cells and anti-FAP@OMF-NPs exhibited marked destruction of HSs containing high FRα/high FAP cells. Recent reviews and clinical trials indicated that, although FAP/CAF-targeted agents can remodel stroma and enhance immune infiltration, their clinical benefit has been modest and limited by CAF heterogeneity 46, as demonstrated in multiple preclinical models and early-phase clinical studies. The humanized anti-FAP monoclonal antibody sibrotuzumab was evaluated in a phase I clinical trial in patients with advanced solid tumors, including colorectal cancer, and showed good tolerability but no objective tumor responses 47. More recent approaches include FAP-targeted immunocytokines in phase II clinical trials 48, as well as FAP-directed antibody-drug conjugates, radionuclide-based imaging and therapy 49, and early-phase FAP-CAR-T cell clinical trial (NCT03932565) have been reported. Together, these additions highlight the rationale and translational relevance of the dual FRα CAR-T and FAP-targeted NP approach presented in this study.

According to transcriptomic and phenotypic profiling, heterogeneity of CAFs in breast cancer was detected 50. The CAF-S1 cells were characterized by high FAP, PDGFRβ, and α-SMA and exhibited potent immunosuppressive activity through recruitment and polarization of regulatory T cells. In contrast, the CAF-S4 subset exhibited low FAP and was associated with extracellular matrix remodeling 51, 52. Importantly, the CAF-S1 signature was correlated with poor prognosis, higher tumor grade, and diminished immune infiltration in TNBC 53. High stromal FAP levels have been consistently linked to increased tumor aggressiveness, immunosuppressive microenvironment, and poorer patient outcomes 53. Additionally, IL-6 derived from FAP-positive CAFs, but not FAP-negative CAFs, activated PD-L1 expression in breast cancer cells attenuating the tumor killing function of CAR-T cells 17. Our therapeutic strategy specially targets the tumor-promoting FAP-high CAF-S1 population, which represents the predominant and clinically relevant CAF subset. The immunohistochemical analyses demonstrated stromal FAP expression in more than 90% of breast cancer cases 54, highlighting the majority of FAP-positive CAFs constitute within the tumor microenvironment. Hence, the anti-FAP antibody functionalized NPs eliminated tumor-promoting FAP-positive CAFs, may have the potential to attenuate cancer progression and PD-L1 expression on cancer cells. By selectively depleting FAP-high CAFs through anti-FAP antibody-functionalized NPs loaded with OMF, our approach aims to eliminate the primary immunosuppressive and tumor-promoting CAF population while potentially reducing PD-L1 expression on cancer cells. In oppose, the limited contribution of FAP-low CAFs to tumor progression and immunosuppression suggested their persistence is unlikely to drive significant treatment resistance.

In our present work, the focus was to establish proof-of-concept for the selective elimination of FAP-overexpressing (High-FAP, including PC-B-130CAFs and -132CAFs), but not Low-FAP CAFs (-004CAFs), using anti-FAP antibody functionalized NPs. Future investigations will need to address this heterogeneity by including a broader panel of patient-derived CAFs and potentially integrating complementary targeting approaches to minimize the risk of escape by FAP-low subpopulations. Thus, targeting FRα using immunotherapy such as CAR-T cells offer selective and effective cytotoxicity delivery to TNBC cells. This approach minimises off-target effects on normal cells with restricted FRα expression. Heightened FAP expression in CAFs suggests its potential as a biomarker for identifying and targeting the TME. Moreover, we observed minimal cytotoxicity of anti-FAP@OMF-NPs against non-FAP CAFs or T lymphocytes in our co-culture assays (data not shown). This is supported by findings that FAP-activated prodrugs and anti-FAP-drug conjugates displayed negligible off-target toxicity in normal tissues and immune compartments due to the limited surface expression of FAP in quiescent fibroblasts and immune cells 55, 56 and the antibody-guided NP internalization mechanism that prevents non-specific OMF diffusion 57. Given CAFs' emerging role in tumor progression and therapy resistance, targeting FAP-expressing CAFs offers novel therapeutic strategies. These strategies could disrupt tumor stroma and enhance anticancer treatment efficacy.

Spheroids have diverse applications ranging from anti-cancer drug testing to functional assays in cancer immunotherapy. In our current study, we focused on developing and characterizing the 3D HS platform as an intermediate preclinical model to assess additive effects of CAR-T cells combined with anti-FAP@OMF-NPs. This system offers several advantages, including controlled CAF:cancer cell ratios, real-time monitoring of tumor-immune interactions, and reduced animal usage in the early proof-of-concept phase. Nevertheless, we fully acknowledge that *in vivo* validation remains essential to evaluate additional parameters such as fibrosis, ECM remodeling, hypoxia, and the pharmacokinetics/pharmacodynamics of CAR-T cell and NPs combinations in a living system. Culturing cancer cells alongside CAFs in 3D models as HOs and HSs overcomes limitations of traditional culture methods. These models more closely resemble the TME. They should therefore yield results that better mimic *in vivo* conditions and patients' physiology 58. The present study was designed as a proof-of-concept investigation to demonstrate selective CAF targeting and establish the mechanistic basis of *in vitro* validation of OMF-induced apoptosis. Our use of the 3D HS model provides a critical intermediate platform that bridges conventional *in vitro* studies and *in vivo* experimentation. This model closely recapitulates the complexity of CAF-immune-tumor organization observed in patient-derived breast cancer tissues. Previous studies have shown that such 3D HSs preserve the histological architecture and transcriptional profiles of patient tumors, including CAF distribution patterns and immune-tumor interface complexity 59, 60. Thus, this platform enables an accurate *ex vivo* assessment of combinatorial CAF- and tumor-directed immunotherapeutic strategies with clinically relevant complexity. We acknowledge that an *in vivo* validation in appropriate murine models represents an essential next step to evaluate the pharmacokinetics, biodistribution, systematic toxicity, and therapeutic efficacy of our anti-FAP NPs. These experiments are needed to ultimately strengthening the translation toward clinical application. In this context, high FRα-expressing MDA-MB-231 cancer cells were co-cultured with high FAP-expressing CAFs at a 1:3 ratio. This approach determined how spatial dimensionality affects FRα-specific CAR-T cells and anti-FAP@OMF-NPs reaction (**Supplementary Movies**) 61, 62. Compared with 2D monolayers, our 3D cultures exhibited more pronounced morphological degradation. This degradation occurred in both cancer cells and CAFs following treatment 63, 64. Taken all together, the HSs used herein represent effective models of high FRα cancer cells and high FAPα stromal fibroblasts. These models accurately reflect the real-world situation of TNBC patient tissues 65.

FRα-specific CAR-T cells were generated from five healthy donor's blood using CAR-carrying lentiviral transduction. Flow cytometry evaluated transduction efficiency. Flow cytometry analysis revealed optimal anti-FRα-CAR protein expression on T cells at 37.61% ± 7.31%. This expression level approximates an earlier report from Luangwattananun and collaborators showing 55.3% ± 6.2% 12. These results demonstrate successful CAR transduction. Furthermore, anti-FRα-CAR-T cells contained higher proportions of cytotoxic T lymphocytes (CTLs, CD3+/CD8+) than helper T cells (Th cells, CD3+/CD4+). The CTL predominance indicates successful generation of anti-tumor CAR-T cells 66.

Consequently, the killing activity of combined FRα-CAR-T cells with anti-FAP@OMF-NPs was investigated in 3D HOs and HSs for 24 h. Fluorescence imaging revealed that anti-FRα-CAR-T cells selectively killed MDA-MB-231 homospheroids (M231 HOs) but not MCF-10A homospheroids (M10A HOs) compared with mock T cells. This selectivity reflects high FRα expression in MDA-MB-231 cells 46. FRα-CAR-T cells exhibited minimal cytotoxicity toward the FRα-negative MCF-10A cells, consistent with the established characterization of MCF-10A as a FRα-negative normal breast epithelial model 12. Similarly, high FAP-expressing PC-B-130CAFs HOs (130CAFs HOs) and PC-B-132CAFs HOs (132CAFs HOs) responded strongly to anti-FAP@OMF-NPs. In contrast, PC-B-004CAFs HOs (004CAFs HOs) and HDFa HOs showed no response. The NP modification strategy successfully improved OMF drug specificity, as previously demonstrated 28.

Following confirmation of target cell killing by both CAR-T cells and anti-FAP@OMF-NPs, combination effects were assessed. The highest percentage of cell lysis occurred in M231/130CAF HSs and M231/132CAF HSs after combined treatment. These HSs received both CAR-T cells and NPs. In contrast, M231/HDFa HSs and M231/004CAF HSs contained only high FRα-expressing cells. In these HSs, the killing effect derived mainly from anti-FRα-CAR-T cells.

In addition, the multiplex cytokine bead array revealed elevated induction and secretion of key cytokines. IFN-γ, granzyme A, granzyme B, and granulysin increased following exposure to anti-FRα-CAR-T cells. These cytokines also increased with combined anti-FRα-CAR-T cell and anti-FAP@OMF-NP treatment. This cytokine release triggered apoptotic cell death in TNBC cells 67. CAFs in breast cancer are well known to contribute to an immunosuppressive tumor microenvironment by secreting soluble mediators, particularly IL-4 and IL-10 68, 69. IL-4 mediates Th2 and M2 macrophage polarization, leading to immunosuppressive TME 70, while IL-10 inhibits the functions of DC and T cells and the induction of Treg 71. Preclinical studies have demonstrated that CAF depletion reduced immunosuppressive IL-10 signaling and ECM density, thereby enhancing T cell infiltration and function 72, 73. To elucidate the mechanistic basis of the additive effect observed with combined anti-FAP@OMF-NP and anti-FRα-CAR-T cell therapy, we performed multiplex cytokine profiling of HS culture supernatant. The cytokine bead array analysis revealed significantly reduced secretion of either IL-4 or IL-10 or both, from HS treated with both anti-FRα-CAR-T cells and anti-FAP@OMF-NPs compared to those from HS treated with CAR-T cells alone across all 3 donors **(Supplement Table S2).** These findings imply that the selective elimination of FAP-positive CAFs by anti-FAP@OMF-NPs directly attenuates the production of key immunosuppressive cytokines within TME. It is therefore plausible that similar mechanisms underlie the additive effects observed in our system. The findings suggest that anti-FRα-CAR-T cells promoted cytokine secretion. This observation aligns with previous work by Lal *et al*. 74. Their study showed mesothelin (MSLN)-specific CAR-T cells enhanced cytolytic activity and increased cytokine production in MSLN-expressing tumor cells 74, 75.

However, cytokine production was not observed in spheroids exposed to anti-FAP@OMF-NPs alone. This finding implies cytokines were produced primarily by activated CAR-T cells after encountering target cancer cells. Anti-FAP@OMF-NPs do not participate in CAR-T cells' cytolytic function. Instead, they contribute by reducing CAF-mediated immune suppression in the TME. So far, our findings demonstrate an additive effect when combining anti-FRα-CAR-T cells with anti-FAP@OMF-NPs. This combination results in effective elimination of cancer-CAF HSs. These results align with previous studies investigating combinatorial approaches using CAR-T cell therapy and NP-based strategies for cancer treatment 76.

## Conclusions

In summary, the study elucidates the mimicry of TNBC patient tissues using high FRα-cancer cells and high FAP-fibroblast spheroids as models *in vitro* to test the effect of target treatment on these two components using anti-FRα-CAR-T cells and anti-FAP@OMF-NPs (**Fig. 6Q**). The successful generation and characterisation of anti-FRα-CAR-T cells demonstrated efficacy in selectively targeting FRα-expressing TNBC cells. The engineered CAR-T cells exhibited significant surface expression of FRα-specific CAR protein. They showed a balanced composition of helper and cytotoxic T cells. These characteristics validate their potential for TNBC therapy. Combination therapy using anti-FRα-CAR-T cells and anti-FAP@OMF-NPs demonstrated enhanced cytotoxicity against TNBC-CAF 3D-HSs. This combination also increased cytokine production (**Fig. 6Q**). The additive effects indicate potential for combining CAR-T cell therapy with NP-based strategies in effective TNBC treatment. This approach modulates the microenvironment surrounding cancer cells whilst enhancing anti-tumor immune responses. These findings underscore the promise of targeted therapies and combination approaches for improving TNBC treatment outcomes.

## Supplementary Material

Supplementary figures and tables, movie legends.

Supplementary movie 1.

Supplementary movie 2.

Supplementary movie 3.

Supplementary movie 4.

Supplementary movie 5.
