# Supplementary material for: Dual Targeting of FAP-Directed Nanoparticles and FRα-Specific CAR-T Cells Induces Additive Anti-Tumor Effects in Triple-Negative Breast Cancer
Source: Int J Biol Sci. 2026 Feb 18;22(5):2736–53. doi: 10.7150/ijbs.122417 (PMC12965329; doi:10.7150/ijbs.122417)
Supplement: Supplementary file 1 — Supplementary figures and tables, movie legends. [file ijbsv22p2736s1.pdf]

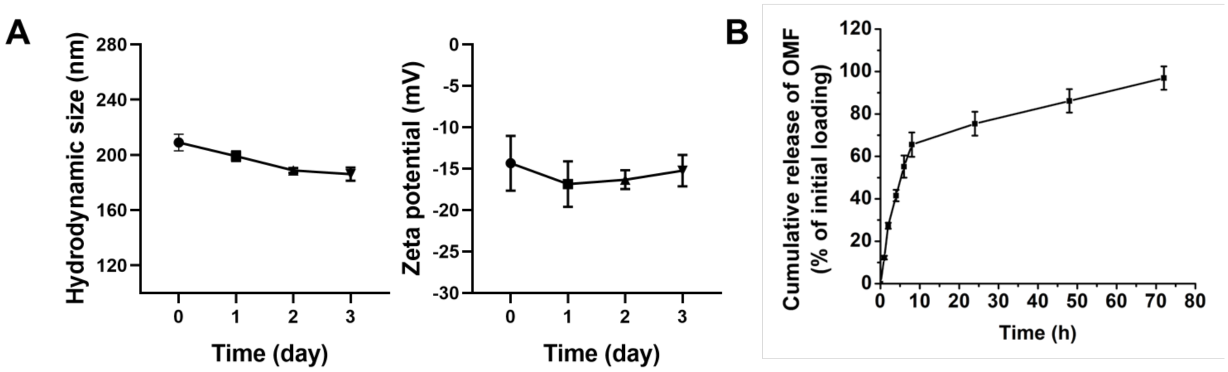

Fig. S1. Stability and drug release profile of Anti-FAP@OMF-NPs. **(A)** Stability of Anti-FAP@OMF-NPs after incubation in complete medium at 37°C for 3 days, evaluated by measuring hydrodynamic size (nm) and zeta potential (mV). **(B)** Cumulative OMF release profile of Anti-FAP@OMF-NPs in PBS (pH 7.4) at 37°C over 72 h.

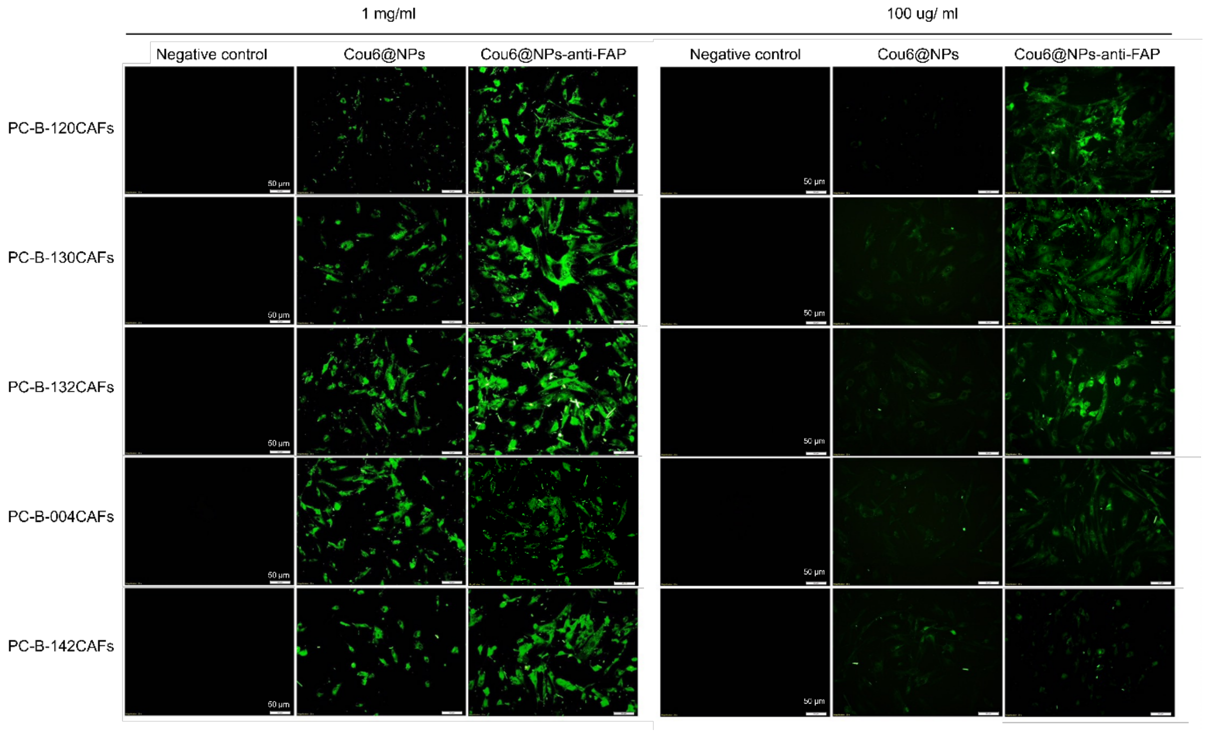

Fig. S2. Internalization efficiency of Cou6@NPs or Cou6@NPs-anti-FAP in FAP-high and FAP-low CAFs. Confocal fluorescence imaging showing cellular uptake of anti-FAP@OMF-NPs (1 mg/mL and 100 μg/mL) in CAF models with differential FAP expression. Green fluorescence signal of Cou6 indicates internalization of NPs in FAP-high CAFs (including PC-B-120CAFs, PC-B-130CAFs, and PC-B-132CAFs) and in FAP-low CAFs (PC-B-140CAFs and PC-B-142CAFs). Scale bars, 50 μm.

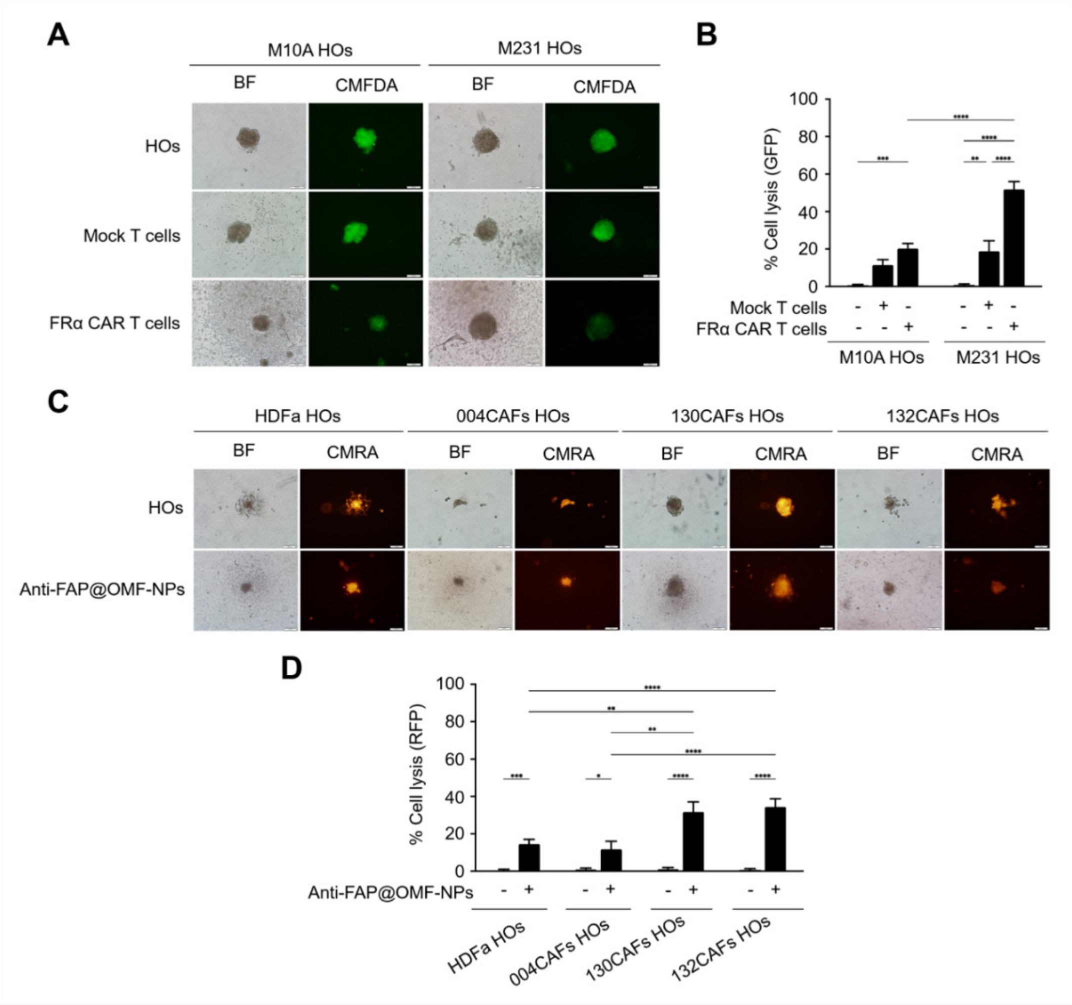

Fig. S3. The representative images and histogram of the % cell lysis of anti-FRα-CAR-T cells (produced from pooled results from HD01 to HD 05) and anti-FAP@OMF-NPs in different spheroids. **(A)** Fluorescence images (green color = cancer) of 3D M10A HOs, and M231 HOs after being treated with anti-FRα-CAR-T cells (produced from pooled results from HD01 to HD 05) for 48 h. **(B)** Percentage of cell lysis of 3D M10A and M231 HOs at 48 h post-treatment. **(C)** Fluorescence images (red color = CAFs) of 3D HDFa HOs, 004CAFs HOs, 130CAFs HOs, and 132CAFs HOs after being treated with anti-FAP@OMF-NPs for 48 h. **(D)** Percentage of cell lysis of 3D HDFa HOs, 004CAFs HOs, 130CAFs HOs, and 132CAFs HOs at 48 h post-treatment. The

results are summarized from five independent experiments (one experiment for one HD, in a total of 5 donors). $*p < 0.05$, $**p < 0.01$, $***p < 0.001$, and $****p < 0.0001$. The scale bar is 100 μm.

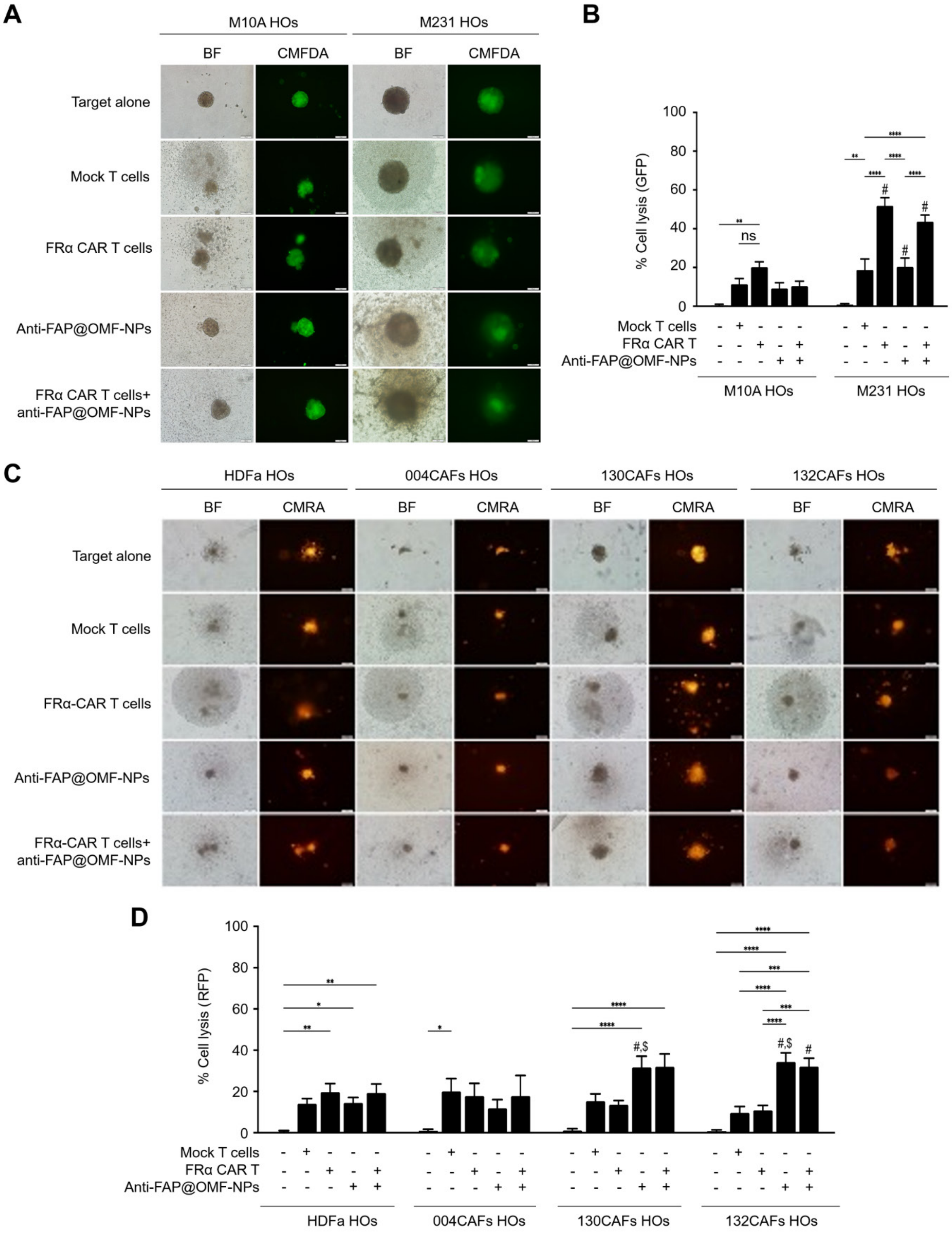
A
M10A HOs
M231 HOs
BF
CMFDA
Target alone
Mock T cells
FRα CAR T cells
Anti-FAP@OMF-NPs
FRα CAR T cells+
anti-FAP@OMF-NPs
B
% Cell lysis (GFP)
Mock T cells
FRα CAR T
Anti-FAP@OMF-NPs
M10A HOs
M231 HOs
ns
C
HDFa HOs
004CAFs HOs
130CAFs HOs
132CAFs HOs
BF
CMRA
Target alone
Mock T cells
FRα-CAR T cells
Anti-FAP@OMF-NPs
FRα-CAR T cells+
anti-FAP@OMF-NPs
D
% Cell lysis (RFP)
Mock T cells
FRα CAR T
Anti-FAP@OMF-NPs
HDFa HOs
004CAFs HOs
130CAFs HOs
132CAFs HOs

Fig. S4. The representative images and histogram of the % cell lysis of anti-FRα-CAR-T cells (produced from pooled results from HD01 to HD 05) and anti-FAP@OMF-NPs in 3D M10A HOs, M231 HOs, HDFa HOs, 004CAFs HOs, 130CAFs HOs, and 132CAFs HOs. **(A)** Fluorescence images (green color = cancer) of different spheroids after being treated with various treatment conditions for 48 h. **(B)** Percentage of cell lysis of 3D M10A and M231 HOs at 48 h post-treatment (# indicates $p < 0.05$ compared to M10A HOs). **(C)** Fluorescence images (red color = CAFs) of different spheroids after being treated with various treatment conditions for 48 h. **(D)** Percentage of cell lysis of 3D HDFa, 004CAFs, 130CAFs, and 132CAFs HOs at 48 h post-treatment (# indicates $p < 0.05$ compared to HDFa HOs and $ indicates $p < 0.05$ compared to 004CAFs HOs). The results are summarized from five independent experiments (one experiment for one HD, in a total of 5 donors). *$p < 0.05$, ***$p < 0.01$, ***$p < 0.001$, and ****$p < 0.0001$. The scale bar is 100 μm.

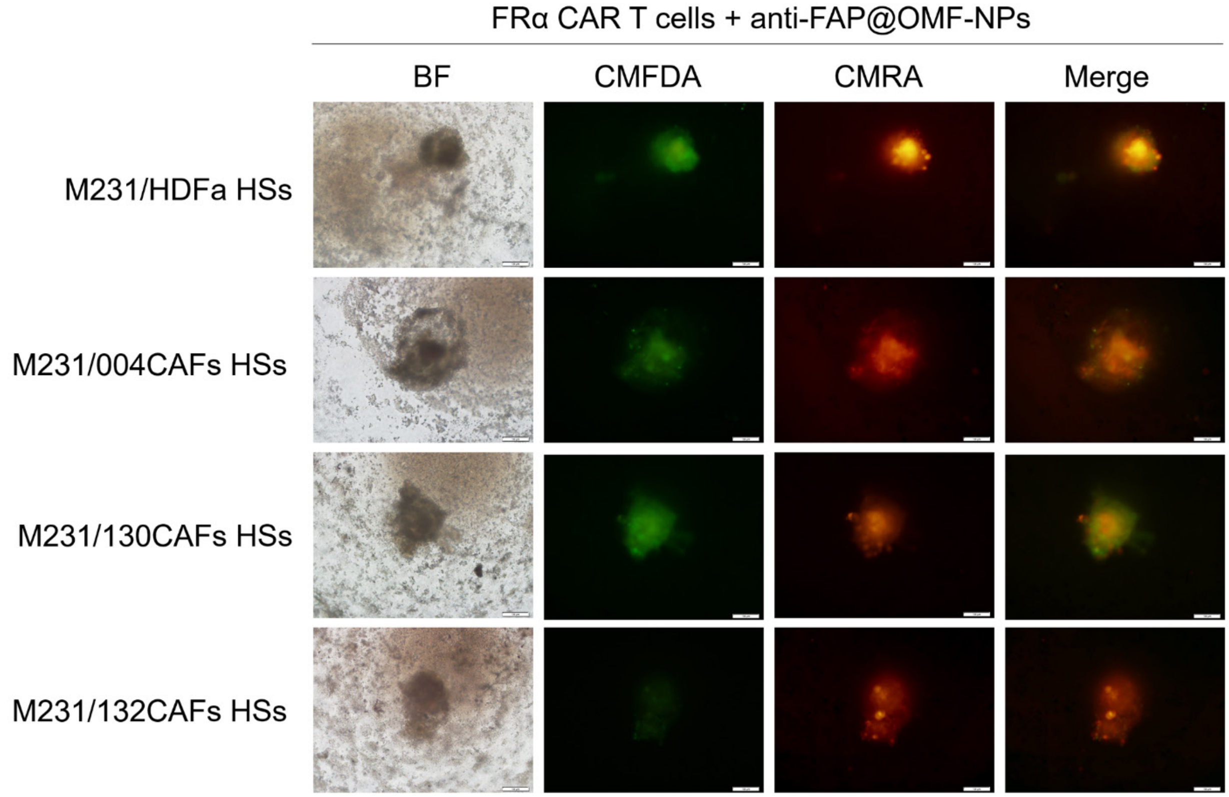

Fig. S5. The representative images of M231/004CAFs HSs, M231/130CAFs HSs, and M231/132CAFs HSs under anti-FRα-CAR-T cells and anti-FAP@OMF-NPs treatment (HD 05). Fluorescence images (green color = cancer and red = CAFs) at 48 h post-treatment. The scale bar is 100 μm.

Supplement Movie S1. The movie of time-lapse imaging of 3D-M231/HDFa HSs, 3D-M231/004CAFs HSs, 3D-M231/130CAFs HSs, and 3D-M231/132CAFs HSs after being treated with anti-FRα-CAR-T cells and anti-FAP@OMF-NPs for 48 h.

Supplement Movie S2. The movie of time of 3D-M231/132CAFs HSs after being treated with anti-FRα-CAR-T cells and anti-FAP@OMF-NPs for 48 h.

Supplement Movie S3. The movie of time of 3D-M231/130CAFs HSs after being treated with anti-FRα-CAR-T cells and anti-FAP@OMF-NPs for 48 h.

Supplement Movie S4. The movie of time-lapse imaging of M231/HDFa HSs during 48 h of combination therapy with anti-FRα-CAR-T cells and anti-FAP@OMF-NPs.

Supplement Movie S5. The movie of time-lapse imaging of M231/004CAF HSs during 48 h of combination therapy with anti-FRα-CAR-T cells and anti-FAP@OMF-NPs.

Supplement Table S1. Physicochemical characterization of the synthesized nanoparticles.

Sample	Hydrodynamic size (nm)	Polydispersity index (PDI)	Zeta potential (mV)	OMF encapsulation efficiency (%)	OMF loading capacity (%)	Antibody conjugation efficiency (%)
OMF-NPs	201.33 ± 3.63	0.10 ± 0.07	-23.30 ± 2.68	-	-	-
Anti-FAP@OMF-NPs	215.17 ± 2.61	0.06 ± 0.02	-14.22 ± 0.49	72.68 ± 1.64	8.26 ± 0.19	58.74 ± 3.94

Hydrodynamic size, polydispersity index (PDI), and zeta potential were measured using a Zetasizer. The percentage of OMF encapsulation efficiency and drug loading capacity were quantified by ultraviolet–visible spectrophotometry, while the percentage of antibody conjugation efficiency was determined using the Bradford protein assay.

Supplement Table S2. Raw data of cytokine bead array analyzed by flow cytometry (pg/ml).

Samples	Cytokine concentration (pg/ml)												
	**IL-2**	**IL-4**	**IL-10**	**IL-6**	**IL-17A**	**TNF-α**	**sFas**	**sFasL**	**IFN-γ**	**Granzyme A**	**Granzyme B**	**Perforin**	**Granulysin**
HD01-M231/130CAFs+Mock	1.0	1.0	404.1	366.4	5377.8	628.5	1299.5	10975.5	6941.1	137706.3	464604.3	11867.6	159707.8
HD01-M231/130CAFs+FRα CAR-T cells	38.4	86.1	199.8	28.9	44912.5	1.0	816.2	5750.4	1827.8	69865.2	364748.5	2462.3	137321.2
HD01-M231/130CAFs+FRα CAR-T cells+Anti-FAP@OMF-NPs	8.6	1.0	1.0	281.4	7358.6	1.0	702.6	3751.1	1512.2	20173.1	219498.8	1100.1	24832.9
HD02-M231/130CAFs+Mock	160.5	319.2	21.9	21.1	23773.1	1.0	679.2	6205.9	4136.3	62005.6	342478.1	13676.2	124032.5
HD02-M231/130CAFs+FRα CAR-T cells	63.2	161.9	224.4	296.8	37340.4	1.0	936.7	8739.2	10380.9	99209.0	365652.7	20186.4	144784.7
HD02-M231/130CAFs+FRα CAR-T cells+Anti-FAP@OMF-NPs	279.7	1.0	256.9	1.0	14520.0	1.0	539.6	1022.0	456.1	969.1	4167.8	80.4	463.9
HD05-M231/130CAFs+Mock	366.7	316.6	565.0	462.1	114645.6	798.1	1523.7	25711.8	18567.5	368927.4	525723.4	113438.9	237988.1
HD05-M231/130CAFs+FRα CAR-T cells	1233.5	424.6	519.1	748.8	122147.4	749.1	1318.0	20161.6	30218.8	342951.7	504608.1	71719.5	158812.7
HD05-M231/130CAFs+FRα CAR-T cells+Anti-FAP@OMF-NPs	571.6	6985.6	83.7	1.0	40366.4	1.0	754.0	3678.7	2315.3	19376.9	47696.8	2011.6	43274.2
